# Hybrid Radiant Disinfection: Exploring UVC and UVB Sterilization Impact on the Mechanical Characteristics of PLA Materials

**DOI:** 10.3390/polym15244658

**Published:** 2023-12-10

**Authors:** Mohamed A. Aboamer, Ahmed S. Alsuayri, Ahmad Alassaf, Tariq M. Alqahtani, Bakheet A. Alresheedi, Ghazwan N. Saijari, Elamir A. Osman, Nader A. Rahman Mohamed

**Affiliations:** 1 Department of Medical Equipment Technology, College of Applied Medical Sciences, Majmaah University, Majmaah 11952, Saudi Arabia; m.aboamer@mu.edu.sa (M.A.A.);; 2 Erada and Mental Health Hospital, Al-Kharj 16246, Saudi Arabia; 3 Department of Preventive Dental Science, College of Dentistry, Majmaah University, Majmaah 11952, Saudi Arabia; 4 Biomedical Technology Department, Inaya Medical Colleges, Riyadh 13541, Saudi Arabia; 5 Biomedical Engineering Department, Faculty of Engineering, Misr University for Science and Technology (MUST), Giza 12568, Egypt

**Keywords:** poly(lactic acid) (PLA), ultraviolet C (UVC) irradiation, ultraviolet B (UVB) irradiation, mechanical properties, hybrid disinfection technique

## Abstract

This study explores the impact of disinfection techniques on the mechanical properties of poly(lactic acid) (PLA), a crucial material in the production of medical implants, tissue engineering, orthopedic devices and drug delivery systems, owing to its biocompatibility and ease of manufacturing. The focus is on evaluating the effectiveness of ultraviolet (UV) type C (254 nm wavelength) and the combined use of type C and B (310 nm wavelength) disinfection methods. Fifteen tensile test specimens (ASTM D638) and fifteen compression test specimens (ASTM D695) were utilized to assess PLA’s mechanical properties, including yield strength, ultimate strength, and fracture strength. The investigation involved subjecting the specimens to the specified disinfection methods and evaluating these properties both before and after the disinfection process. In the tensile test, a statistically significant difference (*p* = 0) in yield displacement was observed among the three groups. Additionally, a notable difference (*p* = 0.047) in fracture displacement was identified between the untreated group and the UVC and UVB combination group. No discernible impact on yield or fracture forces was noted. In the compression test, there was a significant difference (*p* = 0.04) in yield displacement and a clear difference (*p* = 0.05) in fracture force between the untreated group and the UVC and UVB combination group. The hybrid combination of UVC and UVB disinfection techniques did not affect yield force in both tensile and compression tests. However, it demonstrated a clear impact on displacement, suggesting its potential as a promising disinfection technique in the medical field.

## 1. Introduction

### 1.1. Background

#### 1.1.1. Poly(Lactic Acid) in Medical Applications

Poly(lactic acid) (PLA), a biodegradable thermoplastic either derived from renewable sources or synthetic, finds diverse applications in biomedicine [1,2,3,4,5,6,7,8,9,10]. It is utilized for surgical implants, drug delivery systems, tissue engineering, dental applications, sutures, orthopedic devices, wound dressings, diagnostic devices, and medical equipment, showcasing its biocompatibility and versatility.

#### 1.1.2. Tensile Test

ASTM D638, a standardized test by ASTM International, assesses the tensile properties of plastics, crucial for material understanding, design, and quality control. Using a “dog bone” shaped specimen, it measures tensile strength, elongation at break, and modulus of elasticity [11].

#### 1.1.3. Compression Test

ASTM D695, another ASTM International standard, evaluates the compressive properties of rigid plastics. It involves applying a compressive load to cylindrical or rectangular specimens, measuring compressive strength and compressive modulus of elasticity [12].

#### 1.1.4. Ultraviolet C

Ultraviolet C (UVC) radiation, with wavelengths of 100–280 nm, is germicidal, destroying microorganisms. UVC finds applications in water treatment, air purification, surface disinfection, food industry, healthcare, and UV germicidal irradiation. Safety precautions and material considerations are crucial, due to its potential harm [13,14,15,16,17].

#### 1.1.5. Ultraviolet B

UVB radiation (280–315 nm) has both beneficial and harmful effects. Used in controlled exposure for medical treatments like UVB phototherapy, it addresses skin conditions under medical supervision [14,15,16,18,19].

### 1.2. Literature Review

UV light is a type of electromagnetic radiation with a wavelength between 100 and 400 nm. ISO 21348:2007 divides UV radiation into three groups: UVA (315–400 nm), UVB (315–280 nm), and UVC (200–280 nm) [20]. UVA radiation can tan the skin; however, if it is used for a long time, it can be damaging. UVB can be used to treat skin problems such as psoriasis. UVC has been shown to be the most effective way to kill both viruses and bacteria, especially at 254 nm. According to prior research, 3.7 mJ/cm^2^ at 254 nm is the dosage required to inactivate SARS-CoV-2 [1,2,3,4,5]. It has been shown that UVB light has the same impact on DNA as UVC, but only a few studies have examined the bactericidal impact of UVB radiation, especially its effect on oral bacteria, in which 310 nm UVB at doses of 105, 210, 315, and 420 mJ/cm^2^ was investigated [14,15,16,18]. Moreover, few studies have been conducted on the combination of UVC at 254 nm and UVB at 310 nm, which may decrease the disinfection time and affect the mechanical properties of the PLA material as a hybrid radiation.

Mechanical testing is an essential aspect of materials science and engineering that allows for the evaluation of a material’s mechanical properties. Tensile and compression tests are two common mechanical testing methods that are used to determine the strength, elasticity, and other mechanical properties of materials. The fabrication of test specimens is a critical aspect of these tests and can significantly affect the results. Traditional manufacturing methods for producing test specimens involve machining or cutting the material to a specific size, which is time-consuming and expensive. However, in recent years, the fabrication of tensile and compression test specimens using three-dimensional (3D) printing technology has gained significant attention, because of its potential to produce complex geometries with high accuracy and precision. Among the various materials used for 3D printing, PLA has emerged as a popular choice because of its biodegradability, ease of printing, and affordability. Tensile and compression tests are essential for determining the mechanical properties of materials and structures under different loading conditions, and the use of 3D-printed PLA specimens offers several advantages over traditional manufacturing methods.

Additive manufacturing (AM) is a common method for creating different parts. This is carried out using a process called layer-by-layer manufacturing or three-dimensional (3D) printing. In contrast to conventional manufacturing techniques, such as injection molding, bending, and stamping, 3D printing builds objects by combining materials. AM surpasses traditional methods because of its energy-saving capability, low environmental impact, and scalability. Additionally, AM also reduces the amount of waste material, the time required to make a part, and the costs incurred, by making a small number of parts quickly. Because of its possible benefits, AM has been used in fields such as automotives, electronics, aerospace, healthcare monitoring, construction, and fashion [21,22,23,24,25,26]. 

Amza et al. exposed several 3D-printed polymer samples to various doses of UVC radiation that are commonly used in medical sterilization procedures [27]. The mechanical properties of the samples were assessed, and they found that the stiffness and tensile strength of the polymers decreased with increasing exposure to UVC radiation. Additionally, the 3D-printed polymers became more brittle over time, owing to the formation of free radicals and the degradation of polymer chains caused by UVC radiation. The polymers used in the study were acrylonitrile butadiene styrene (ABS), PLA, and polyethylene terephthalate glycol (PETG). ABS is a thermoplastic polymer commonly used in 3D printing because of its high strength and durability. PLA is a biodegradable polymer made from renewable resources and is commonly used in food packaging and biomedical applications. PETG is a durable, transparent polymer used in medical devices, water bottles, and other applications in which transparency and impact resistance are important [27]. This study provides valuable insights into the impact of UVC radiation on the mechanical properties and aging of 3D-printed polymers. These findings highlight the importance of considering the effects of sterilization methods on the performance of materials used in medical applications. The three polymers investigated in this study are commonly used in various industries, including the medical and biomedical fields [27].

Aboamer et al. investigated the mechanical properties of ABS specimens using ASTM 638, 695, and 790 [11,12,28], while also exploring the effects of UVC radiation as a sterilization method [17]. The study involved using fused deposition modeling to create 30 specimens with 30% filling for tensile, compression, and bending tests. Half of the specimens were treated with UVC radiation, while the other half were not. A dosage of 13.5 J/cm^2^ with an exposure time of 48 min, equivalent to 3650 sterilization treatments or 10 years of sterilization, was selected. The average ultimate stress for the tensile, compression, and bending tests was 34.5 ± 7.4, 25.4 ± 0.5, and 24.5 ± 2.1 MPa, respectively. The analysis of variance test indicated that UVC radiation had a significant impact on the tensile specimens, with a *p*-value of 0.012, which was below the significance threshold of 0.05, resulting in the rejection of the null hypothesis.

Aboamer et al. examined the impact of UVB radiation on the degradation and oxidation of low-density polyethylene (LDPE) and high-density polyethylene (HDPE) specimens, which are reliable, cost-effective biomaterials and alternatives to bone transplants [29]. The study utilized ASTM 638 and 695 to evaluate the tensile and compression tests of the 20 HDPE and 20 LDPE specimens. Sterilization was performed at a dosage of 13.5 J/cm^2^ for 48 min. The results revealed that LDPE exhibited a lesser effect on mechanical tensile properties compared to HDPE, with the *p*-value of yield stress equaling 3.008 × 10^−4^, the *p*-value of ultimate stress equaling 2.5 × 10^−4^, and the *p*-value of break stress equaling 0.0075.

The combination of UVC and UVB radiation has been reported to be more effective in destroying bacteria than UVC or UVB radiation alone. When UVC and UVB are combined, they have an additive effect on the inactivation of microorganisms because they induce the same photochemical reactions on DNA. However, the combination of UVA with UVC/UVB or the sequential application of UVA after UVC/UVB treatment reduced the inactivation of *E. coli*. This is because UVA has DNA repair and photoreactivation effects that can counteract the effects of UVC and UVC/UVB [19].

While previous works have explored the impact of irradiation on the mechanical properties of PLA materials, we believe our study adds value by investigating not only the effects of UVC but also the combination of UVC and UVB as a hybrid disinfection technique. Earlier studies have suggested UVB at 310 nm as a potential disinfection tool, challenging the notion that only UVC at 254 nm can effectively kill bacteria and viruses. Our study stands out as the first to focus on this hybrid combination of UVC and UVB as a disinfection technique for PLA material.

This study aims to investigate the disinfection effect of this hybrid combination of UVC and UVB on the mechanical properties of PLA, which can be used in the production of medical implants, tissue engineering, orthopedic devices and drug delivery systems, owing to its biocompatibility and ease of manufacturing. Both UVC and UVB radiation can destroy the most well-known bacteria, and the combination of UVB and UVC radiation is still not popular, although it may decrease the disinfection exposure time in future investigations. 

### 1.3. Problem Statement

There is a pressing need to investigate the disinfecting impact of combining two different types of UV light, types C and B, on the mechanical characteristics of PLA, which is suitable for use in the production of medical device frames. While UVB and UVC demonstrated a promising effect in destroying bacteria [19], the hybrid combination of these two types of radiation is not typically employed but can potentially shorten the amount of time that bacteria are exposed to the disinfection process.

### 1.4. Objectives of the Study

The primary objectives of this research study revolve around the investigation of the effects of UVC and UVC + UVB treatment on the mechanical properties of PLA materials. To achieve this, the study aims to provision a UVC light source emitting at a wavelength of 254 nm and a UVB light source with a wavelength of 310 nm, facilitating controlled exposure conditions. Furthermore, the research will utilize a 3D printer to fabricate a total of 30 test specimens, with 15 dedicated to tensile testing following ASTM D638 standards, and the remaining 15 designed for compression testing in accordance with ASTM D695 guidelines. By conducting these experiments, the study seeks to provide valuable insights into how UVC and UVC + UVB treatment impact the mechanical properties of PLA materials, which will be analyzed using statistical tools. These findings hold significance for a wide range of applications, particularly in materials science and engineering, with potential implications for the development of more resilient and adaptable PLA-based products.

## 2. Materials and Methods

**Figure 1:**
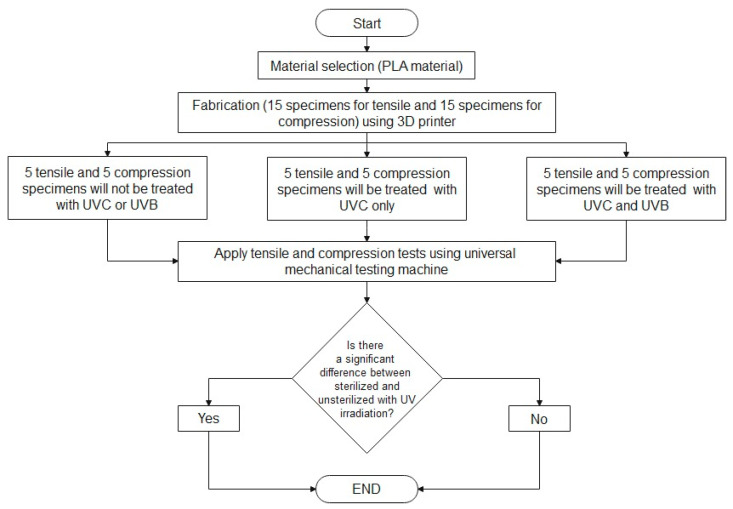
The proposed method for the whole study.

The proposed method is divided into five major sections, as shown in Figure 1. The first is material selection, where the PLA material has been chosen for two reasons: first, it has a reasonable cost, and second, its availability in the market. The PLA material was provided from CREALITY (Shenzhen Creality 3D Technology Co., Shenzhen, China) for use in the 3D printer, and it comes with the following specifications: CR-PLA White, 1.75 mm diameter, 1.0 kg weight, and a recommended printing temperature range of 195–220 °C. The second section describes the fabrication of the specimens using a 3D printer based on the ASTM 638 and ASTM 695 specimen sizes for tensile and compression tests. Thirty specimens were included in this investigation. The specimens were divided into three groups: the first group was not treated with UVC or UVB irradiation; the second group was treated with UVC only; and the third group was treated with UVC and UVB. A mechanical testing machine was used to test the mechanical properties of the PLA before and after UV treatment. Finally, an analysis of variance (ANOVA) was employed to determine whether there was a significant difference between the treated and untreated specimens.

### 2.1. Three-Dimensional Printer

A 3D printer is a computer-controlled machine that creates three-dimensional objects by adding materials layer-by-layer, a process known as additive manufacturing. These machines have revolutionized various industries by enabling rapid prototyping, custom manufacturing, and the production of complex geometries, which would be challenging or impossible to achieve using traditional manufacturing methods.

#### 2.1.1. Working Principle

3D printing follows the basic principle of adding material layer by layer to build an object. This contrasts with subtractive manufacturing methods, such as machining, in which the material is removed from a solid block to create the final shape.

#### 2.1.2. Materials

3D printers can work with a wide range of materials, including plastics, metals, ceramics, composites, and even biological materials such as living cells. The choice of material depends on the specific applications and capabilities of the 3D printer.

#### 2.1.3. Types of Three-Dimensional Printing Technologies

There are several 3D printing technologies available, each with its own advantages and limitations. Common 3D printing technologies include fused deposition modeling (FDM) and fused filament fabrication (FFF), which are among the most popular and affordable 3D printing technologies. A heated nozzle is used to extrude a thermoplastic filament that solidifies as it cools.

- Stereolithography (SLA): SLA uses a UV laser to selectively solidify liquid resins layer by layer. This method produces highly detailed and accurate parts.
- Selective laser sintering (SLS): SLS uses a high-power laser to sinter (fuse) powdered materials, such as plastics or metals, into solid objects.
- Digital light processing (DLP): Similar to SLA, DLP uses a light source (often a projector) to cure liquid resin. This method is known for its speed and accuracy.

**Figure 2:**
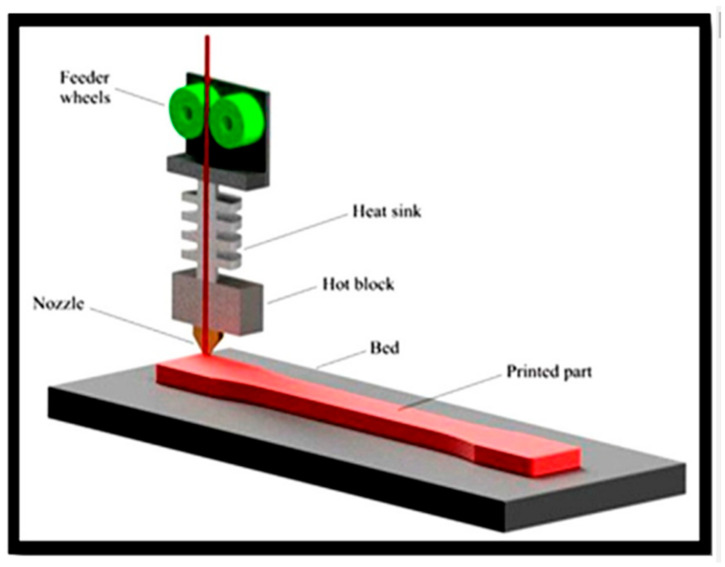
Schematic of the FDM printing process.

3D printing has numerous applications in various industries, including aerospace, automotive, healthcare, architecture, fashion, and consumer goods. Common applications include rapid prototyping, custom medical implants, architectural models, and the production of intricate components. Figure 2 depicts the FDM process. Fiber deposition printing (FDP) is a well-known technology for creating structural components using polymer filaments with varying characteristics. Polymeric materials with different flexibilities, operating temperatures, and hardness values can also be employed. FDP can use several materials, the most common of which are PLA and ABS. In several investigations, short and long fiber-reinforced thermal polymers were used as feedstock for the FDP method [30,31,32]. In Ref. [32], for example, carbon-fiber-reinforced plastic composite specimens have been created using FDP, carbon fibers, and ABS. FDM was used to 3D-print the samples using a TRONXY X5SA-500PRO machine (Shenzhen Tronxy Technology Co., Ltd, Shenzhen, China). Table 1 and Table 2 provide the filament specifications and qualities, as well as the printing settings.

### 2.2. Geometrical Data of Tensile and Compression Specimens

#### 2.2.1. Tensile Specimens

**Figure 3:**
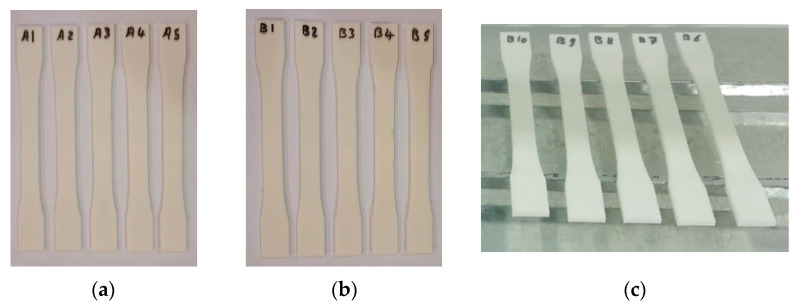
Tensile test specimens (ASTM D638): (**a**) untreated specimens (A1, A2, A3, A4 and A5), (**b**) treated specimens with UVC (B1, B2, B3, B4 and B5), and (**c**) treated specimens with UVC and UVB (B6, B7, B8, B9 and B10).

Tensile test specimens were manufactured in accordance with ASTM D638 under a regulated ambient temperature of 25 °C. Fifteen 3D-printed ASTM D638 specimens were produced, as illustrated in Figure 3. Tensile specimens were fabricated to conform to the ASTM D638 standard with the following average dimensions: an inner length of 57 ± 0 mm, outer length of 173 ± 0.06 mm, width of 19 ± 0.04 mm, and thickness of 3.2 ± 0.03 mm.

These fifteen ASTM D638 specimens were divided into three groups: group one, which remained untreated by UV irradiation (A1, A2, A3, A4, and A5); group two, which underwent treatment with UVC only (B1, B2, B3, B4, and B5); and the third group, which received treatment with both UVC and UVB irradiation (B6, B7, B8, B9, and B10).

#### 2.2.2. Compression Specimens 

**Figure 4:**
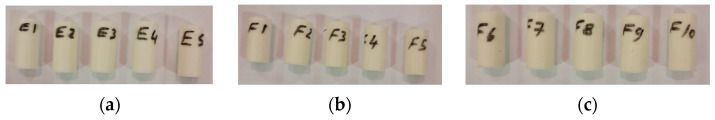
Compression test specimens (ASTM D695): (**a**) untreated specimens (E1, E2, E3, E4 and E5), (**b**) specimens treated with UVC (F1, F2, F3, F4 and F5), and (**c**) specimens treated with UVC and UVB (F6, F7, F8, F9 and F10).

Compression test specimens were manufactured in accordance with ASTM D695 at a regulated ambient temperature of 25 °C. Fifteen ASTM D695 specimens were produced, as depicted in Figure 4. Compression specimens were fabricated to follow the ASTM D695 standard with the following average dimensions: a length of 25.3 ± 0 mm and a diameter of 12.5 ± 0 mm.

These fifteen ASTM D695 specimens were divided into three groups: group one, which remained untreated by UV irradiation (E1, E2, E3, E4, and E5); group two, which underwent treatment with UVC only (F1, F2, F3, F4, and F5); and the third group, which received treatment with both UVC and UVB irradiation (F6, F7, F8, F9, and F10).

Based on ASTM D638 and ASTM D695, a tensile speed of 2 mm/min and a frequency of 20 Hz were employed in the data collection; the test speed for the compression test specimen was 5 mm/min, and the frequency was 20 Hz.

In addition, tensile tests were performed on a universal testing machine (UTM) with a 5-kN load cell. The tensile load F [kN] and upper crossbar movement L [mm] were recorded and saved. Based on these measurements, it was possible to determine the loads in MPa and the nondimensional strains as follows:

(1) $$\delta =\frac{F}{A},\;\;\epsilon =\frac{\Delta L}{L_{0}}$$

where δ is the stress measured in (kN/mm^2^) or (kPa), *L*_0_ is the starting length in (mm), *F* is the force in (kN), *A* is the cross-sectional area in (mm^2^), and ΔL is the change in length in (mm) [29].

### 2.3. Effectiveness in Fighting Germs

**Figure 5:**
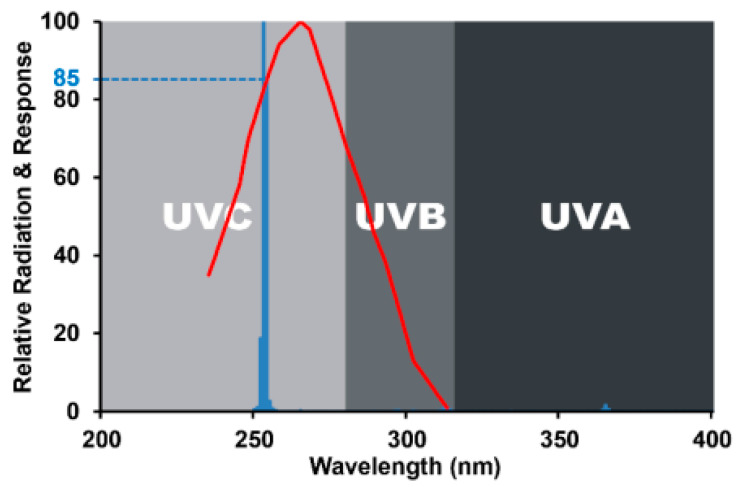
Effect of different wavelengths on germs.

In terms of viral removal, not all UVC wavelengths are equally effective. The degradation of DNA and RNA was detected at approximately 265 nm (red lines). This wavelength is close to the maximum UVC discharge lamp output (254 nm, blue line). As shown in Figure 5, UVC discharge lamps emit approximately 85% of the theoretical maximum germicidal effectiveness [19].

#### 2.3.1. UV Irradiation Enclosure

**Figure 6:**
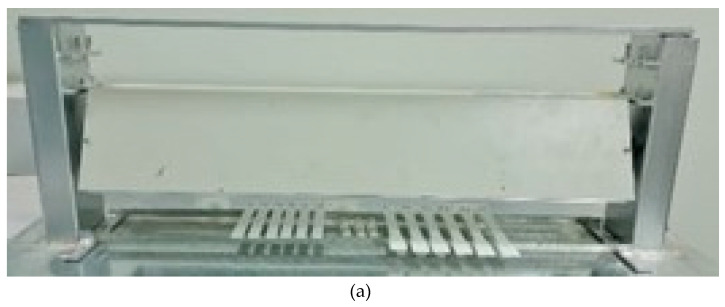

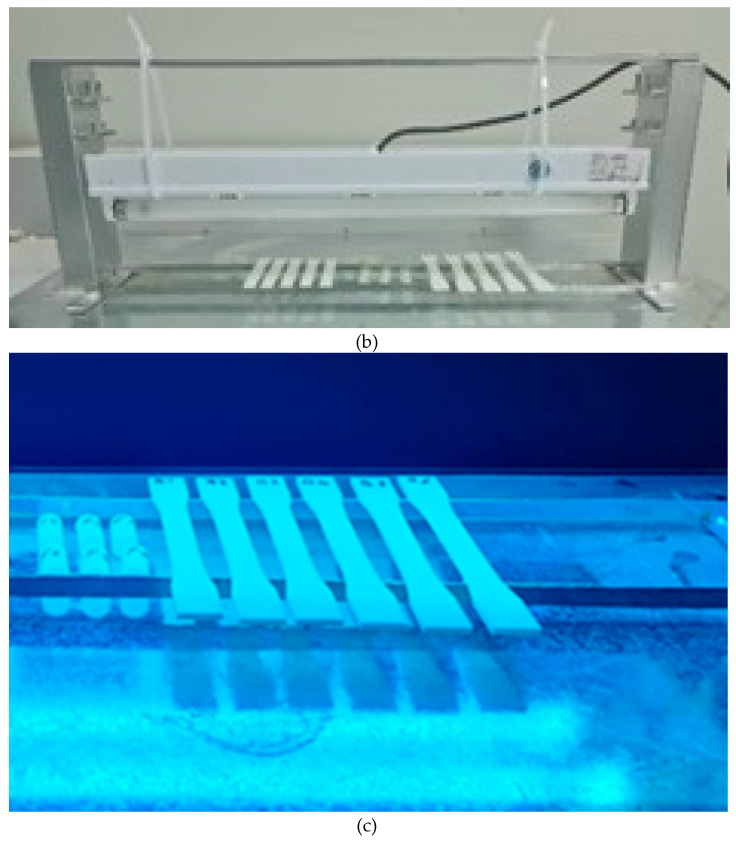
UV irradiation enclosure: (**a**) the adjustable locally made enclosure and the irradiation lamps, (**b**) the arrangement of specimens and the wireless data logger, and (**c**) the setup of the samples inside the UV radiation container.

For UV radiation exposure, a locally made box with two 20 W UV lights at a wavelength of 254 nm was used. The container could hold a space of 8–16 cm between the surfaces of the two lamps and the surfaces of the samples. Figure 6a shows a locally made movable cage and irradiation lamps with dimensions of 24 L × 6 W × 4.25 H in (610 × 152 × 108 mm) and a weight of 10 lb (4.5 kg). With the help of a UV radiometer that came with the ILT tools [23], the radiation levels in the entire treated area were recorded. The device allows users to change the lamps, as shown in Figure 6b, which shows another type of UV lamp using a power of 20 W and the same dimensions as the UVC lamps. Figure 6c shows the setup of the samples inside the UV radiation container, where a portable data logger was used to monitor the temperature and humidity during treatment [33].

#### 2.3.2. Exposure Time Calculations

The UV light-emitting robot used two 20 W UVC lamps, and the distance between the lamps and the untreated surfaces was maintained at 12 cm. According to previous investigations, SARS-CoV-2 can be sufficiently inactivated at a dose of 3.7 mJ/cm^2^ at 254 nm. In addition, a recent study [34] found that a minimum exposure duration of 30 min is required for 48 W of power to achieve complete disinfection.

The following equation [35] can be used to determine the system exposure duration, which is measured in seconds:

(2) $$t=\left(2\pi_{Lr}D\right)∕P$$

where *L* is the length of the UVC lamp in cm, *r* is the distance in cm, *D* is the UVC dosage in mJ/cm^2^, and *P* is the power emitted by the UVC light in W. Following the completion of the necessary calculations, the required dose is 3.7 mJ/cm^2^. The length of the UVC lamp was 55 cm, the total wattage was 40 W, and the distance between the lamps and the untreated surfaces was 8 cm. Calculations indicated that the required exposure time should be more than 48 min; therefore, 1 h was selected as the exposure time for this experiment. In addition, a UVB lamp was used as a treatment tool for 48 × 2 h, which equivalent to UVC treatment.

### 2.4. One-Way ANOVA Test

ANOVA, or analysis of variance, is a statistical technique used to analyze and compare the means of three or more groups or treatments to determine if there are statistically significant differences between them. It is a powerful tool for understanding variations in data and is commonly used in various fields, including science, engineering, social sciences, and business. 

A one-way ANOVA is a special form of the linear model. It was performed as follows:

(3) $$y_{ij}=\propto_{j}+\varepsilon_{ij}$$

where:

- $y_{ij}$ is an observation, where i is the number of observations and *j* is a different group of variable *y*.
- $\propto_{j}$ shows the average number of populations in the *j*th group.
- $\varepsilon_{ij}$ is the random error, which is independent, normally distributed, has a mean of zero, and a range that remains the same.

## 3. Results and Discussion

### 3.1. Universal Testing Machine

The UTM is a versatile mechanical testing device designed to assess the mechanical properties of various materials and components. This section provides an overview of UTM’s significance, operation, and applications.

UTMs are indispensable tools in the fields of materials science, engineering, and quality control. They enable the evaluation of critical mechanical characteristics, including tensile strength, compression strength, flexural strength, and hardness. These properties are vital for product design, material selection, and ensuring the safety and reliability of the manufactured items. 

**Figure 7:**
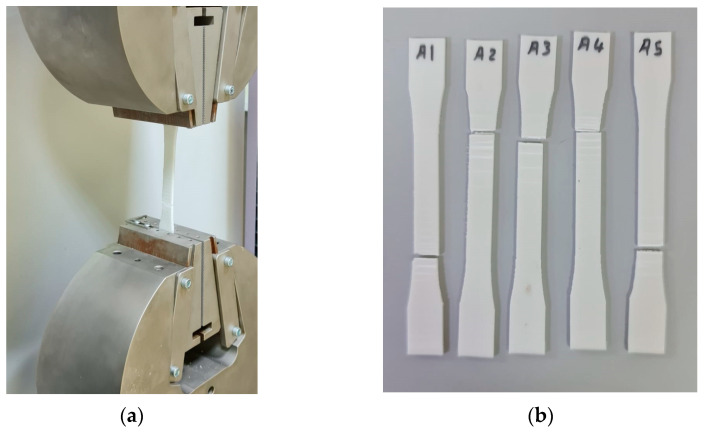

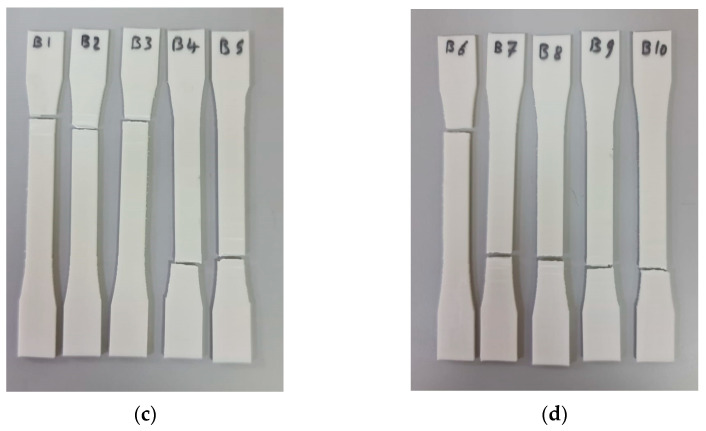
Tensile test: (**a**) universal testing machine (UTM), (**b**) untreated tensile specimens (A1, A2, A3, A4 and A5), (**c**) UVC-treated tensile specimens (B1, B2, B3, B4 and B5), and (**d**) tensile specimens treated with UVC and UVB (B6, B7, B8, B9 and B10).

A UTM was used to determine the tensile parameters, such as yield, ultimate, and fracture stresses and forces, for three different groups of tensile specimens based on ASTM D638, as shown in Figure 7a. Figure 7b–d show the tensile specimens before treatment, after UVC treatment, and after UVB and UVC treatment, respectively. 

**Figure 8:**
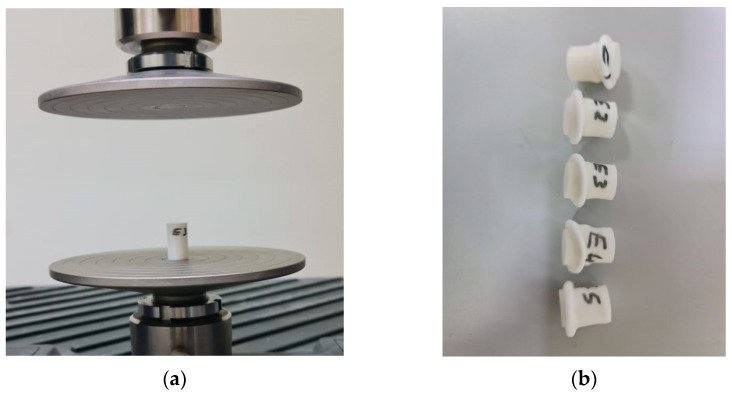

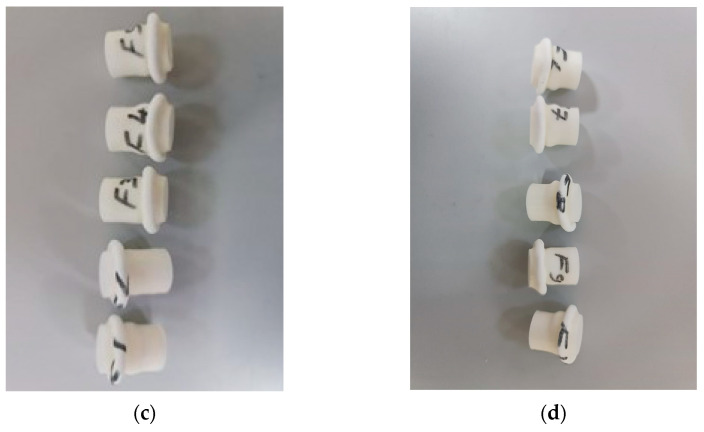
Compression test: (**a**) UTM, (**b**) untreated compression specimens with UV (E1, E2, E3, E4 and E5), (**c**) treated compression specimens with UVC (F1, F2, F3, F4 and F5), and (**d**) treated compression specimens with UVC and UVB (F6, F7, F8, F9 and F10).

A UTM was used to determine the compression parameters, such as the yield, ultimate, and fracture stresses and forces, for the three different groups of compression specimens based on ASTM D695, as shown in Figure 8a. Figure 8b–d show the compression specimens before treatment, after UVC treatment, and after UVB and UVC treatment, respectively.

### 3.2. Tensile Test

#### 3.2.1. Before UV Radiation

**Figure 9:**
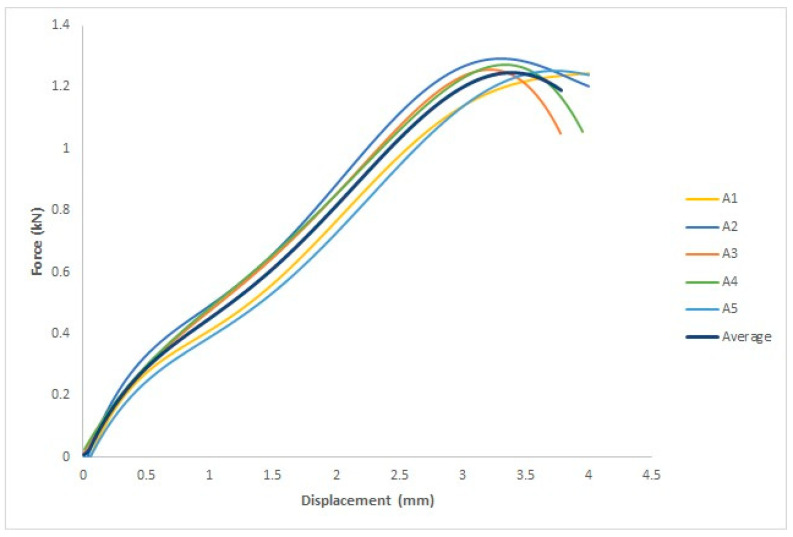
Force–displacement curves for the five specimens and the average curve.

This study aims to determine whether there are any changes in the mechanical properties of specimens treated by UVC irradiation, specimens treated with UVC and UVB, and untreated specimens. Five specimens were employed for each group, and the force–displacement curve for each specimen and the average of the five specimens are illustrated in Figure 9, where the *x*-axis represents the displacement in mm and the *y*-axis represents the force in kN.

Based on the ASTM D638 standard, four parameters were measured: yield force in kN, fracture force in kN, yield displacement in mm, and fracture displacement in mm. 

As shown in Table 3, the four parameters are the measured yield force in kN, fracture stress in kN, yield displacement in mm, and fracture displacement in mm. For the first specimen, the yield force was 1.2 kN, and the yield displacement was 3.4 mm. For the second specimen, the yield force was 1.3 kN, and the yield displacement was 3.4 mm. For the third specimen, the yield force was 1.2 kN, and the yield displacement was 3.4 mm. For the fourth specimen, the yield force was 1.3 kN, and the yield displacement was 3.4 mm. For the fifth specimen, the yield force was 1.2 kN, and the yield displacement was 3.4 mm. For the first specimen, the fracture force was 1.2 kN, and the fracture displacement was 4 mm. For the second specimen, the fracture force was 1.2 kN, and the fracture displacement was 4 mm. For the third specimen, the fracture force was 1 kN, and the fracture displacement was 3.8 mm. For the fourth specimen, the fracture force was 1.1 kN, and the fracture displacement was 4 mm. For the fifth specimen, the fracture force was 1.2 kN, and the fracture displacement was 4 mm. 

#### 3.2.2. After UVC Irradiation

**Figure 10:**
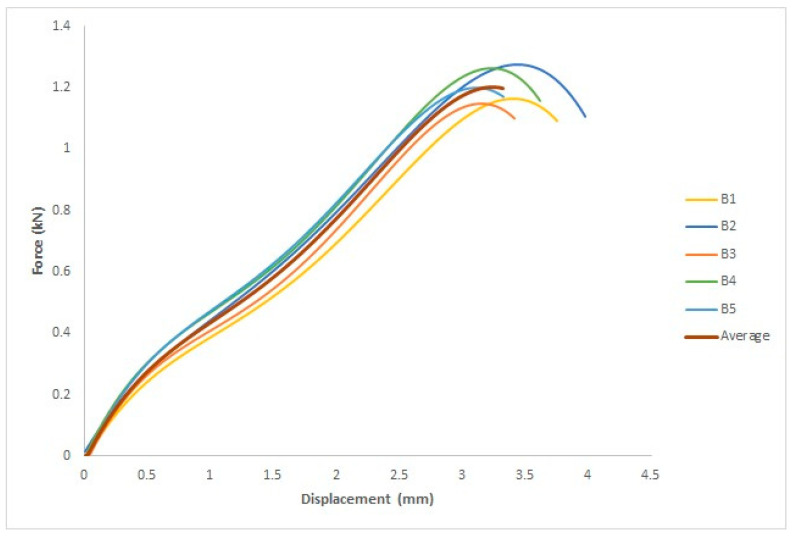
Force–displacement curve for the five specimens treated with UVC and the average curve.

The force–displacement curve for each specimen and the average of the five specimens are depicted in Figure 10, where the *x*-axis represents the displacement in mm and the *y*-axis represents the force in kN.

The ASTM D638 standard was used to quantify four parameters: yield force in kN, fracture stress in kN, yield displacement in mm, and fracture displacement in mm.

Table 4 shows the four measured characteristics: yield force in kN, fracture stress in kN, yield displacement in mm, and fracture displacement in mm. The first specimen had a yield force of 1.2 kN and a yield displacement of 3.2 mm. The yield force for the second specimen was 1.3 kN, and the yield displacement was 3.2 mm. The third specimen had a yield force of 1.1 kN and a yield displacement of 3.2 mm. The fourth specimen had a yield force of 1.3 kN and a yield displacement of 3.2 mm. The fifth specimen had a yield force of 1.2 kN and a yield displacement of 3.2 mm. The fracture force for the first specimen was 1.1 kN, and the fracture displacement was 3.8 mm. The fracture force for the second specimen was 1.1 kN, and the fracture displacement was 4 mm. The fracture force for the third specimen was 1.1 kN, and the fracture displacement was 3.4 mm. The fourth specimen had a fracture force of 1.2 kN and a fracture displacement of 3.6 mm. The fifth specimen had a fracture force of 1.2 kN and a fracture displacement of 3.3 mm.

#### 3.2.3. After UVC and UVB Irradiation

**Figure 11:**
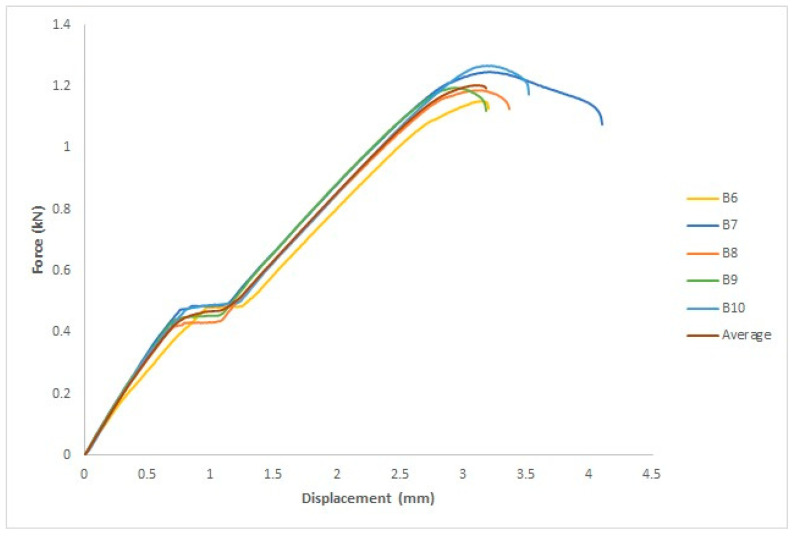
Force–displacement curve for the five specimens treated with UVC and UVB radiation and the average curve.

Five specimens were used for each group. Figure 11 shows the force–displacement curve for each specimen as well as the average of the five specimens. The *x*- and *y*-axes represent displacement in mm and force in kN, respectively.

Four parameters were assessed based on the ASTM D638 standard: yield force in kN, fracture stress in kN, yield displacement in mm, and fracture displacement in mm.

Table 5 displays the four parameters: yield force (measured in kN), fracture stress (measured in kN), yield displacement (measured in mm), and fracture displacement (measured in mm). The first specimen had a yield displacement of 3.2 mm and a yield force of 1.1 kN. The yield force and displacement of the second specimen were 1.2 kN and 3.1 mm, respectively. The third specimen exhibited a yield displacement of 3.1 mm and a yield force of 1.2 kN. The fourth specimen exhibited a yield displacement of 3.1 mm and a yield force of 1.2 kN. The fifth specimen had a yield displacement of 3.1 mm and a yield force of 1.3 kN. For the first specimen, the fracture displacement was 3.2 mm and the fracture force was 1.1 kN. The second specimen had a fracture displacement of 4.1 mm and a fracture force of 1.1 kN. The third specimen had a fracture displacement of 3.4 mm and a fracture force of 1.1 kN. The fourth specimen exhibited a fracture displacement of 3.2 mm and a fracture force of 1.1 kN. The fifth specimen had a fracture displacement of 3.5 mm and a fracture force of 1.2 kN.

#### 3.2.4. ANOVA Analysis for Tensile Test Specimens

In ANOVA analysis, the blue line represents the control group against which all other groups are compared. If a group is represented by a red line, it indicates a significant difference compared to the control group. Conversely, a gray line signifies that there was no significant difference between the respective group and the control group.

**Figure 12:**
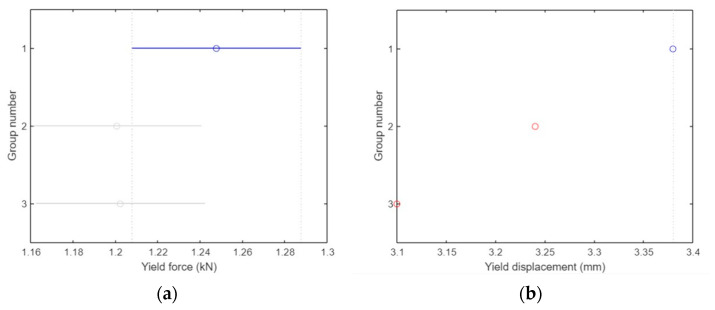

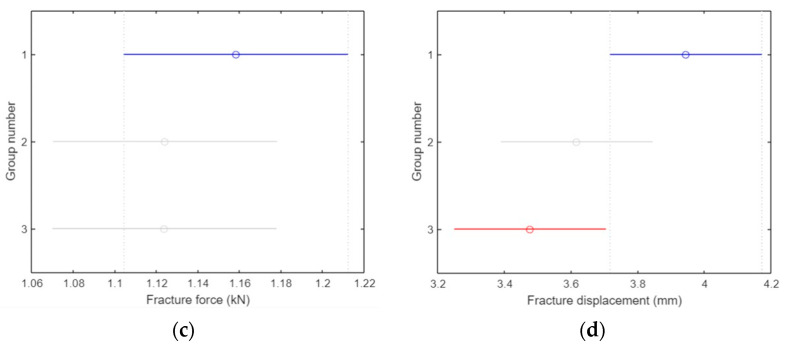
ANOVA analysis for tensile test specimens. (**a**) No groups have means significantly different from group 1. (**b**) Two groups have means significantly different from group 1. (**c**) No groups have means significantly different from group 1. (**d**) The means of group 1 and 3 are significantly different.

For the yield force (kN), there was no significant difference between group 1 and the other two groups (*p* = 0.245), where group 1 refers to specimens that were not treated with any type of UV irradiation, group 2 refers to the group treated with UVC irradiation, and group 3 refers to the group treated with UVC and UVB, as shown in Figure 12a. The *y*-axis represents the three groups and the *x*-axis represents the yield force values in (kN).

As shown in Figure 12b, there was a significant difference in the yield displacement (mm) between group 1 and the other two groups (*p* = 1.2372 × 10^−23^). The *y*-axis represents the three groups, and the *x*-axis represents the yield displacement values in (mm).

As shown in Figure 12c, there was no significant difference in fracture force (kN) between group 1 and the other two groups (*p* = 0.6). 

As illustrated in Figure 12d, groups 1 and 3 showed significant differences (*p* = 0.047) in fracture displacement (mm). The three groups are shown on the *y*-axis, whereas the fracture displacement values in (mm) are shown on the *x*-axis.

#### 3.2.5. Average of Stress–Strain

**Figure 13:**
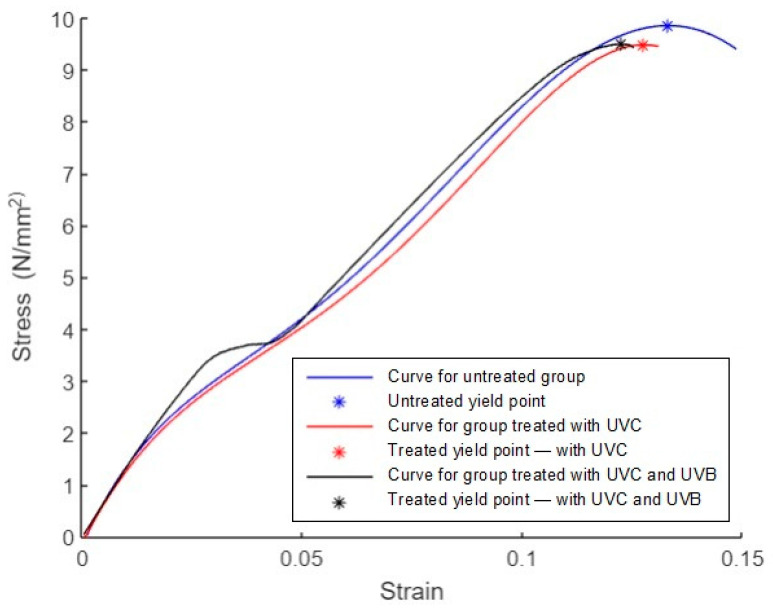
Average stress–strain curves for tensile specimens.

The average curves for groups 1, 2, and 3 are shown in Figure 13. As mentioned in the ASTM D638 standard, the tensile yield point is the first point on the stress–strain diagram where a rise in strain occurs without a rise in stress [11]. The yield points are marked with * symbols. The blue curve corresponds to the untreated group with a yield stress of 9.8 N/mm^2^, a yield strain of 0.13, a fracture stress of 9.4 N/mm^2^, and a fracture strain of 0.15. The red curve corresponds to the group treated with UVC irradiation, with a yield stress of 9.5 N/mm^2^, a yield strain of 0.13, a fracture stress of 9.4 N/mm^2^, and a fracture strain of 0.13. The black curve corresponds to the group treated with UVC and UVB irradiation, with a yield stress of 9.5 N/mm^2^, a yield strain of 0.12, a fracture stress of 9.4 N/mm^2^, and a fracture strain of 0.13.

### 3.3. Compression

#### 3.3.1. Before UV Irradiation

**Figure 14:**
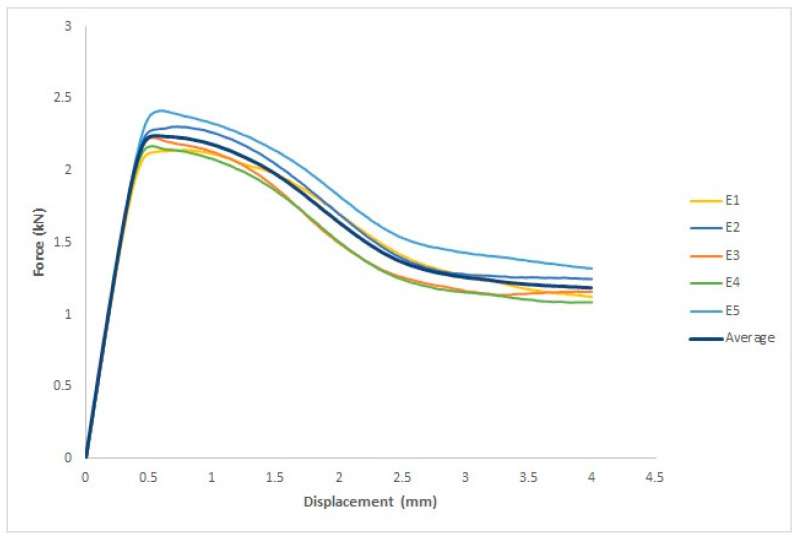
Force–displacement curves for the five untreated specimens and average curve.

Five specimens were used for each group. The force–displacement values obtained from each individual specimen and the average values derived from the five specimens are shown in Figure 14. The *x*-axis of the graph represents the displacement measured in millimeters (mm), whereas the *y*-axis represents the force measured in kilonewtons (kN). The ASTM D965 standard includes the measurement of four parameters: the yield force in kilonewton (kN), fracture force in kilonewton (kN), yield displacement in millimeters (mm), and fracture displacement in millimeters (mm) [12].

The four parameters examined were the yield force in kN, fracture stress in kN, yield displacement in mm, and fracture displacement in mm, as listed in Table 6. The first specimen had a yield force of 2.1 kN and a yield displacement of 0.78 mm. The yield force of the second specimen was 2.3 kN, and the yield displacement was 0.73 mm. The third specimen had a yield force of 2.2 kN and a yield displacement of 0.53 mm. The fourth specimen had a yield force of 2.2 kN and a yield displacement of 0.53 mm. The fifth specimen had a yield force of 2.4 kN and a yield displacement of 0.61 mm. The fracture force for the first specimen was 1.1 kN, and the fracture displacement was 4 mm. The fracture force for the second specimen was 1.2 kN, and the fracture displacement was 4 mm. The fracture force for the third specimen was 1.2 kN, and the fracture displacement was 4 mm. The fourth specimen had a fracture force of 1.1 kN and a fracture displacement of 4 mm. The fifth specimen had a fracture force of 1.3 kN and a fracture displacement of 4 mm.

#### 3.3.2. After UVC Irradiation

**Figure 15:**
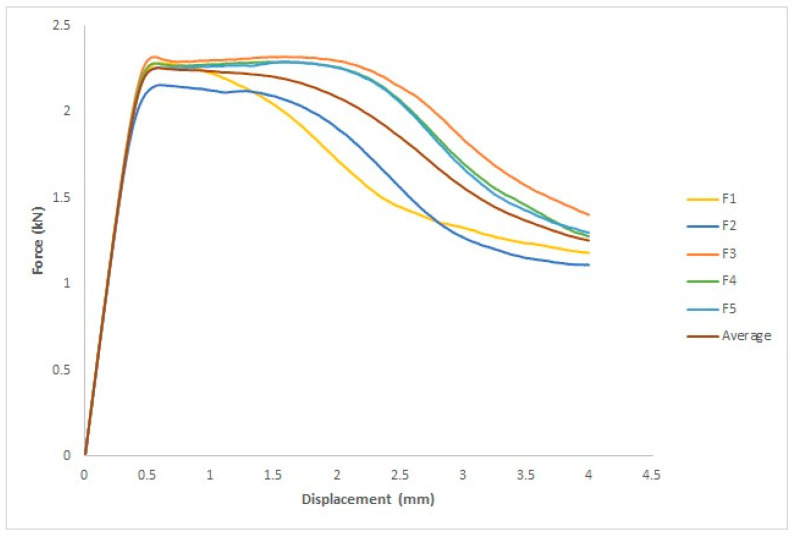
Force–displacement curves for treated five specimens by UVC and average curve.

Figure 15 depicts the force–displacement curve for each specimen and the average of the five specimens, where the *x*-axis represents the displacement in mm and the *y*-axis represents the force in kN. The ASTM D695 standard was used to measure four parameters: yield force (kN), fracture stress (kN), yield displacement (mm), and fracture displacement (mm).

The four parameters tested are listed in Table 7: yield force in kN, fracture stress in kN, yield displacement in mm, and fracture displacement in mm. For the first specimen, the yield force was 2.3 kN and the yield displacement was 0.61 mm. The yield force and displacement of the second specimen were 2.2 kN and 0.59 mm, respectively. The yield force of the third specimen was 2.3 kN, and the yield displacement was 1.6 mm. The yield force of the fourth specimen was 2.3 kN, and the yield displacement was 1.6 mm. The yield force of the fifth specimen was 2.3 kN, and the yield displacement was 1.6 mm. The first specimen had a fracture force of 1.2 kN and a fracture displacement of 4 mm. The fracture displacement of the second specimen was 4 mm, and the fracture force was 1.1 kN. The third specimen had a fracture force of 1.4 kN and a fracture displacement of 4 mm. The fourth specimen exhibited a fracture displacement of 4 mm and a fracture force of 1.3 kN. The fifth specimen exhibited a fracture displacement of 4 mm and a fracture force of 1.3 kN.

#### 3.3.3. After UVC and UVB Irradiation

**Figure 16:**
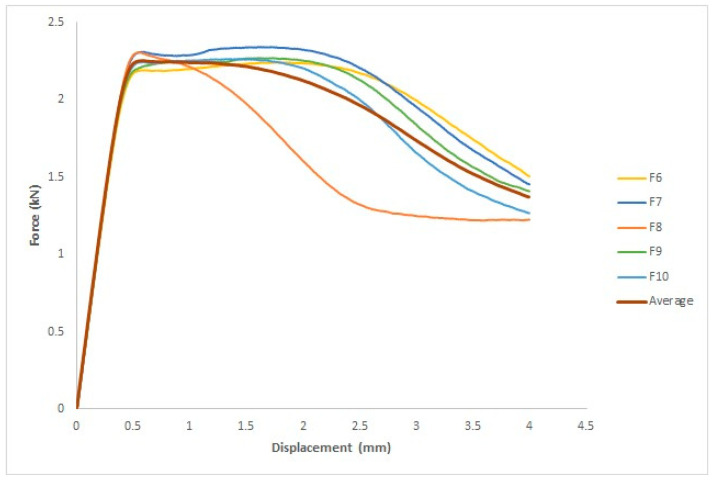
Force–displacement curves for the five specimens treated with UVC and UVB and the average curve.

Five specimens were used for each group. Figure 16 shows the force–displacement curve for each specimen and the average of the five values. The *x*- and *y*-axes measure the displacement in millimeters and force in kilograms, respectively. The ASTM D695 standard was used to evaluate four parameters: yield force in kN, fracture stress in kN, yield displacement in mm, and fracture displacement in mm.

The four measurements are listed in Table 8: yield force (kN), fracture stress (kN), yield displacement (mm), and fracture displacement (mm). For the first specimen, the yield displacement was 1.8 mm, and the yield force was 2.2 kN. For the second specimen, the yield force and displacement are 2.3 kN and 1.7 mm, respectively. The yield displacement of the third specimen was 0.55 mm and the yield force was 2.3 kN. The yield displacement of the fourth specimen was 1.6 mm, and the yield force was 2.3 kN. The yield displacement of the fifth specimen was 1.3 mm, and the yield force was 2.3 kN. The fracture displacement of the first specimen was 4 mm, and the fracture force was 1.5 kN. The fracture displacement of the second specimen was 4 mm, and the fracture force was 1.5 kN. The fracture displacement of the third specimen was 4 mm, and the fracture force was 1.2 kN. The fracture displacement of the fourth specimen was 4 mm, and the fracture force was 1.4 kN. The fracture displacement of the fifth specimen was 4 mm, and the fracture force was 1.3 kN.

#### 3.3.4. ANOVA Analysis for Compression Test Specimens

In ANOVA analysis, the blue line represents the control group against which all other groups are compared. If a group is represented by a red line, it indicates a significant difference compared to the control group. Conversely, a gray line signifies that there was no significant difference between the respective group and the control group.

**Figure 17:**
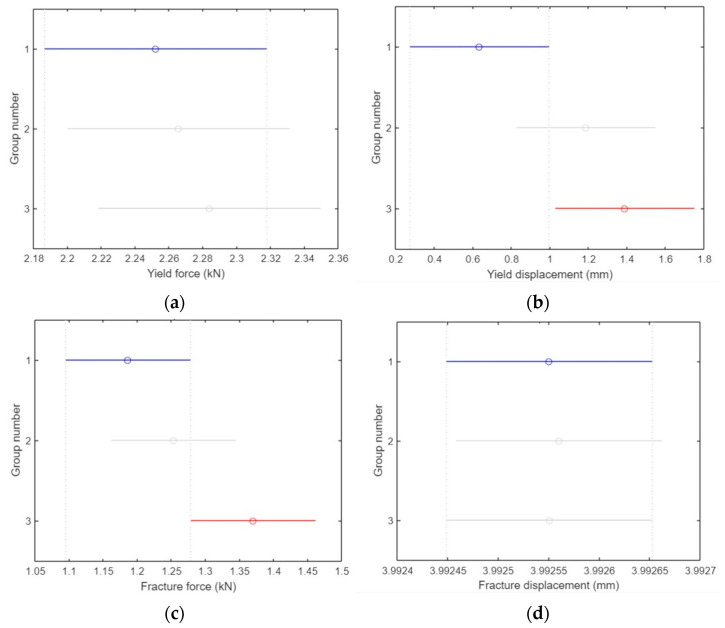
ANOVA analysis for compression test specimens. (**a**) No groups have means significantly different from group 1. (**b**) The means of group 1 and 3 are significantly different. (**c**) The means of group 1 and 3 are significantly different. (**d**) No groups have means significantly different from group 1.

There was no significant difference in the yield force (kN) between groups 1, 2, and 3 (*p* = 0.8), where group 1 refers to specimens that were not treated with any type of UV irradiation, group 2 refers to the group that was treated with UVC irradiation, and group 3 refers to the group that was treated with both UVC and UVB, as shown in Figure 17a. The three groups are represented on the *y*-axis, and the yield force values in (kN) are shown on the *x*-axis.

Figure 17b shows that there was a considerable difference in the yield displacement (mm) between groups 1 and 3 (*p* = 0.04), but no difference was observed between groups 1 and 2. The three groups are shown on the *y*-axis, and the yield displacement values in (mm) are shown on the *x*-axis.

As shown in Figure 17c, there was no significant difference in fracture force (kN) between groups 1 and 2, but there was a clear difference between groups 1 and 2 (*p* = 0.05).

As illustrated in Figure 17d, none of the groups had means significantly different from those of group 1 (*p* = 0.99) in terms of fracture displacement (mm). The three groups are shown on the *y*-axis, whereas the fracture displacement values in (mm) are shown on the *x*-axis.

#### 3.3.5. Average of Stress–Strain

**Figure 18:**
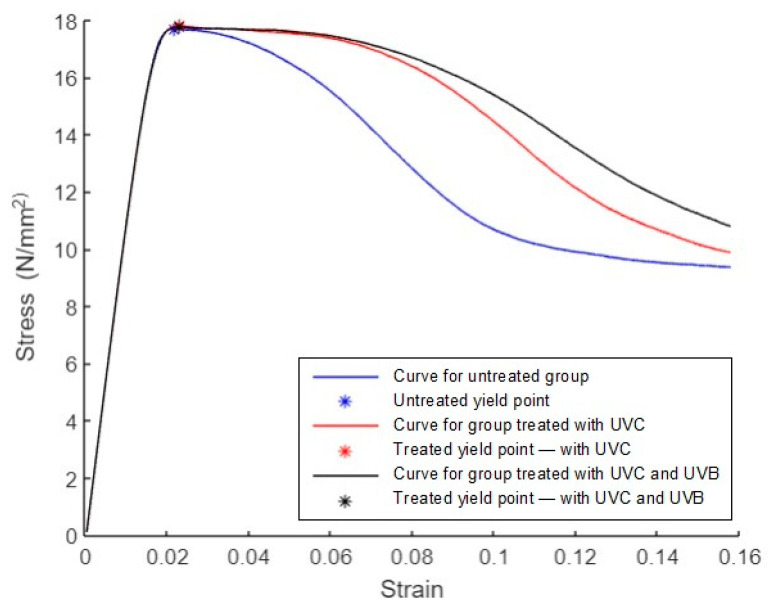
Average stress–strain curves for compression specimens.

The average curves for groups 1, 2, and 3 are shown in Figure 18. As mentioned in the ASTM D695 standard, the compression yield point is the first point on the stress–strain diagram where a rise in strain occurs without a rise in stress. The yield points are marked with * symbols. The blue curve corresponds to the untreated group, with a yield stress of 18 N/mm^2^, a yield strain of 0.02, a fracture stress of 9 N/mm^2^, and a fracture strain of 0.16. The red curve corresponds to the group treated with UVC irradiation, with a yield stress of 18 N/mm^2^, a yield strain of 0.02, a fracture stress of 10 N/mm^2^, and a fracture strain of 0.16. The black curve corresponds to the group treated with UVC and UVB irradiation, with a yield stress of 18 N/mm^2^, a yield strain of 0.02, a fracture stress of 11 N/mm^2^, and a fracture strain of 0.16. 

## 4. Conclusions

This study was divided into two parts: tensile and compression tests. The tensile tests were classified into three categories. The first group was untreated, with no UV irradiation on the test samples, whereas the second and third groups were treated with UV irradiation. The second group was treated with UVC only, and the third group was treated with UVC and UVB. In terms of yield force, no groups were significantly different from the first group; in terms of yield displacement, there was a clear difference between the three groups, indicating that UVC can affect the PLA material; in terms of yield displacement, UVC and UVB also had a clear effect; in terms of fracture force, no groups were significantly different from the untreated group; and in terms of fracture displacement, only group three (treated by UVC and UVB) was significantly different. As a result, UVC and UVB have a noticeable influence on the yield displacement but have no effect on the yield force. Furthermore, the hybrid combination of UVC and UVB has an influence on the fracture displacement but does not affect the fracture force. 

Based on the average stress–strain curve, the first group had the greatest yield value compared to the other two groups, as well as the largest area under the curve, indicating maximum toughness. The compression results were classified into three major groups. The first group was untreated, with no UV irradiation on the test samples, whereas the second and third groups were treated with UV irradiation. The second group was treated with UVC only, and the third group was treated with UVC and UVB. In terms of yield force, no group means were significantly different from the first group. In terms of yield displacement, the means of groups 1 and 3 were significantly different, indicating that the hybrid combination of UVC and UVB can affect the PLA material. In terms of fracture force, the means of groups 1 and 3 were significantly different, indicating that the hybrid combination of UVC and UVB can affect the PLA material. Furthermore, the hybrid combination of UVC and UVB influenced the yield displacement but had no effect on the fracture force and displacement. The average stress–strain curve shows that all groups have the same yield value and the largest area under the curve, indicating maximum toughness.

## Figures and Tables

**Table 1:** Filament specifications and properties.

Diameter (mm)	1.75
Density (g/cm^3^)	1.04
Tensile strength at yield (MPa)	43
Elongation at break (%)	22

**Table 2:** Printing parameters.

Infill Ratio (%)	30
Nozzle diameter (mm)	0.4
Printing temperature (°C)	220
Printing speed (mm/s)	20
Printing pattern rectangular	Grid
Layer height (mm)	0.3

**Table 3:** Tensile test parameters before UV irradiation treatment.

Parameters	A1		A2		A3		A4		A5	
	Disp. (mm)	Force (kN)	Disp. (mm)	Force (kN)	Disp. (mm)	Force (kN)	Disp. (mm)	Force (kN)	Disp. (mm)	Force (kN)
Yield	3.4	1.2	3.4	1.3	3.4	1.2	3.4	1.3	3.4	1.2
Fracture	4	1.2	4	1.2	3.8	1	4	1.1	4	1.2

**Table 4:** Tensile test settings after UVC irradiation.

Parameters	B1		B2		B3		B4		B5	
	Disp. (mm)	Force (kN)	Disp. (mm)	Force (kN)	Disp. (mm)	Force (kN)	Disp. (mm)	Force (kN)	Disp. (mm)	Force (kN)
Yield	3.2	1.2	3.2	1.3	3.2	1.1	3.2	1.3	3.2	1.2
Fracture	3.8	1.1	4	1.1	3.4	1.1	3.6	1.2	3.3	1.2

**Table 5:** Tensile test conditions after using UVC and UVB irradiation.

Parameters	B6		B7		B8		B9		B10	
	Disp. (mm)	Force (kN)	Disp. (mm)	Force (kN)	Disp. (mm)	Force (kN)	Disp. (mm)	Force (kN)	Disp. (mm)	Force (kN)
Yield	3.2	1.1	3.1	1.2	3.1	1.2	3.1	1.2	3.1	1.3
Fracture	3.2	1.1	4.1	1.1	3.4	1.1	3.2	1.1	3.5	1.2

**Table 6:** Compression test parameters before UV irradiation treatment.

Parameters	E1		E2		E3		E4		E5	
	Disp. (mm)	Force (kN)	Disp. (mm)	Force (kN)	Disp. (mm)	Force (kN)	Disp. (mm)	Force (kN)	Disp. (mm)	Force (kN)
Yield	0.78	2.1	0.73	2.3	0.53	2.2	0.53	2.2	0.61	2.4
Fracture	4	1.1	4	1.2	4	1.2	4	1.1	4	1.3

**Table 7:** Compression test settings after UVC irradiation.

Parameters	F1		F2		F3		F4		F5	
	Disp. (mm)	Force (kN)	Disp. (mm)	Force (kN)	Disp. (mm)	Force (kN)	Disp. (mm)	Force (kN)	Disp. (mm)	Force (kN)
Yield	0.61	2.3	0.59	2.2	1.6	2.3	1.6	2.3	1.6	2.3
Fracture	4	1.2	4	1.1	4	1.4	4	1.3	4	1.3

**Table 8:** Compression test settings after UVC and UVB irradiation.

Parameters	F6		F7		F8		F9		F10	
	Disp. (mm)	Force (kN)	Disp. (mm)	Force (kN)	Disp. (mm)	Force (kN)	Disp. (mm)	Force (kN)	Disp. (mm)	Force (kN)
Yield	1.8	2.2	1.7	2.3	0.55	2.3	1.6	2.3	1.3	2.3
Fracture	4	1.5	4	1.5	4	1.2	4	1.4	4	1.3

## Data Availability

The data presented in this study are available on request from the corresponding author.
